# A mRNA landscape of bovine embryos after standard and MAPK-inhibited culture conditions: a comparative analysis

**DOI:** 10.1186/s12864-015-1448-x

**Published:** 2015-04-10

**Authors:** Bas Brinkhof, Helena TA van Tol, Marian JA Groot Koerkamp, Frank M Riemers, Sascha G IJzer, Kaveh Mashayekhi, Henk P Haagsman, Bernard AJ Roelen

**Affiliations:** Department of Farm Animal Health, Faculty of Veterinary Medicine, Utrecht University, Yalelaan 104, Utrecht, 3584 CM The Netherlands; University Medical Center Utrecht, Molecular Cancer Research, PO Box 85060, Utrecht, 3508 AB The Netherlands; Department of Clinical Sciences of Companion Animals, Faculty of Veterinary Medicine, University Utrecht, Yalelaan 108, Utrecht, 3584 CM The Netherlands; BioTalentum Ltd, Aulich L u.26, Gödöllő, 2100 Hungary; Department of Infectious Diseases and Immunology, Faculty of Veterinary Medicine, Utrecht University, Yalelaan 1, Utrecht, 3584 CL The Netherlands

**Keywords:** Pluripotency, Cattle, Morula, Blastocyst, ICM, Trophectoderm, NANOG, MAPK

## Abstract

**Background:**

Genes and signalling pathways involved in pluripotency have been studied extensively in mouse and human pre-implantation embryos and embryonic stem (ES) cells. The unsuccessful attempts to generate ES cell lines from other species including cattle suggests that other genes and pathways are involved in maintaining pluripotency in these species. To investigate which genes are involved in bovine pluripotency, expression profiles were generated from morula, blastocyst, trophectoderm and inner cell mass (ICM) samples using microarray analysis. As MAPK inhibition can increase the *NANOG/GATA6* ratio in the inner cell mass, additionally blastocysts were cultured in the presence of a MAPK inhibitor and changes in gene expression in the inner cell mass were analysed.

**Results:**

Between morula and blastocyst 3,774 genes were differentially expressed and the largest differences were found in blastocyst up-regulated genes. Gene ontology (GO) analysis shows *lipid metabolic process* as the term most enriched with genes expressed at higher levels in blastocysts. Genes with higher expression levels in morulae were enriched in the *RNA processing* GO term. Of the 497 differentially expressed genes comparing ICM and TE, the expression of *NANOG*, *SOX2* and *POU5F1* was increased in the ICM confirming their evolutionary preserved role in pluripotency. Several genes implicated to be involved in differentiation or fate determination were also expressed at higher levels in the ICM. Genes expressed at higher levels in the ICM were enriched in the *RNA splicing* and *regulation of gene expression* GO term. Although *NANOG* expression was elevated upon MAPK inhibition, *SOX2* and *POU5F1* expression showed little increase. Expression of other genes in the MAPK pathway including *DUSP4* and *SPRY4,* or influenced by MAPK inhibition such as *IFNT*, was down-regulated.

**Conclusion:**

The data obtained from the microarray studies provide further insight in gene expression during bovine embryonic development. They show an expression profile in pluripotent cells that indicates a pluripotent, epiblast-like state. The inability to culture ICM cells as stem cells in the presence of an inhibitor of MAPK activity together with the reported data indicates that MAPK inhibition alone is not sufficient to maintain a pluripotent character in bovine cells.

**Electronic supplementary material:**

The online version of this article (doi:10.1186/s12864-015-1448-x) contains supplementary material, which is available to authorized users.

## Background

In mammals, early life starts with the formation of a zygote as a result of the fertilization of an oocyte. Sequential cleavage divisions lead to the formation of a morula stage embryo wherein a fluid-filled cavity emerges called the blastocoel. Two differentiated groups of cells can be distinguished in the embryo that is now called a blastocyst. A group of cells adjacent to the blastocoel, the inner cell mass (ICM), is able to contribute to all cells of the three germ layers and is therefore referred to as being pluripotent. The other group of cells, called the trophectoderm (TE), forms an epithelium surrounding the blastocoel and the ICM and is important for implantation within the uterus and contributes to the non-maternal part of the placenta. In the ICM further differentiation occurs by the formation of the epiblast, that will form the foetus, and the formation of extra-embryonic primitive endoderm (PE) contributing to the yolk sac.

Studies with mouse embryos have advanced our understanding of how a pluripotent cell population is established during pre-implantation development [1-4]. During the first differentiation, the transcription factors CDX2 and OCT4 are key regulators for the formation of respectively TE and ICM. CDX2 represses the activity of OCT4 in mouse TE [5] and is virtually absent in ICM cells [6]. OCT4 in turn can counteract CDX2 activity in the inner cells of the morula. The second differentiation is indicated by the expression of either NANOG or GATA6 in ICM cells fated to become the epiblast or PE respectively [7]. Like for CDX2 and OCT4 in the morula, NANOG and GATA6 inhibit each other’s transcription [4]. Whether the same genes and signalling pathways are also involved in the formation of a pluripotent cell population in other mammals remains to be established. Indeed, in contrast to the mouse, OCT4 protein remains present in the TE of bovine blastocysts even after transcription is down-regulated [8] and its expression is not negatively regulated by CDX2 [9]. In mouse embryos it has been established that GATA6-stimulated fibroblast growth factor (FGF) signalling via the extracellular signal-regulated protein kinase (ERK) is responsible for NANOG repression and thereby the formation of primitive endoderm [2,10-12]. In bovine and human embryos however, although GATA6 expression is specific for primitive endoderm, inhibition of ERK signalling had a more moderate (bovine) or no (human) effect on the numbers of NANOG and GATA6 expressing cells suggesting that in these species other pathways are involved in the formation of the pluripotent cell population [13-15]. These findings suggest species-specific mechanisms active in the specification of ICM, TE, epiblast and PE lineages and that further insight is needed into the molecular basis of cell sorting during the two first differentiation events.

When mouse ICM cells are cultured under defined conditions, their pluripotent character can be maintained [16,17]. However, the establishment of such embryonic stem (ES) cells has only been successful for mice, non-human primates [18], humans [19] and rats [20]. Although pluripotency refers to the capacity to give rise to all embryonic and adult cell types, including the germ line, various states of pluripotency have been described. These states are referred to as “naïve” and “primed”, with “primed” being more developmentally restricted [21]. In mammals other than primates and rodents, the correct stages of embryos that contain pluripotent cells and culture conditions that maintain pluripotency have yet to be established [22].

In order to identify genes that may be important for the acquisition and maintenance of pluripotency in bovine embryos a genome-wide gene expression analysis was performed in morulae, intact blastocysts, TE and ICM. Analyses of gene expression patterns in pre-implantation embryos to distinguish between pluripotent cells of the ICM versus those of the TE have previously made use of cell lines because of the technical difficulties of separating ICM from TE [23,24]. Here we have manually dissected individual ICMs from TE. As the late ICM is composed of both pluripotent epiblast cells and the PE, the pluripotent character of the ICM was enhanced by inhibition of the ERK-pathway resulting in an increased percentage of ICM cells that express *NANOG*.

## Results

### Gene expression profile of pre-implantation embryos

**Figure 1:**
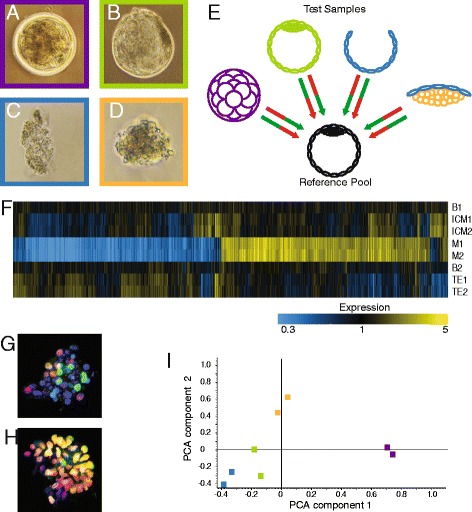
Microarray set-up and sample validation. RNA from morula (**A**, purple), blastocyst (**B**, green), trophectoderm (**C**, blue) and inner cell mass (**D**, orange) was hybridized on microarrays. A common reference sample composed of blastocysts was hybridized with each sample in duplicate in balanced dye-swap (**E**, arrows indicate an array and the used fluorescent label; Cy3: green; Cy5: red). The heat map (**F**) illustrates pairwise clustering of microarray sample replicates. Yellow colour represents over-expressed probes and blue colour represents under-expressed probes as indicated by the colour legend (B = blastocyst; ICM = inner cell mass; M = morula; TE = trophectoderm). Representative pictures of immunofluorescent labelling of dissected ICM (**G**) and TE (**H**) for GATA6 (green) and CDX2 (red); double GATA6-CDX2 nuclei appear yellow. Nuclear staining by DAPI (blue). A 2D principal component analysis plot (**I**) with sample position indicating clustering of trophectoderm (blue), blastocyst (green), inner cell mass (orange) and morula (purple) replicates.

To identify genes involved in bovine pluripotency, gene expression profiles of morula and blastocyst embryos, ICM and TE were generated using microarray analysis. Bovine cumulus oocyte complexes (COCs) were in vitro matured, fertilized and cultured for 5 or 9 days to obtain morula and blastocyst stage embryos, respectively (Figure 1A,B). In addition, ICM and TE were manually dissected from day 9 blastocysts (Figure 1C,D). From all samples RNA was isolated and only those with a RNA integrity number (RIN) ≥8.0 were used for further analysis. To compensate for biological and technical errors, two biological replicates of each sample were labelled with either Cy3 or Cy5 and hybridized on the arrays with a common reference pool consisting of blastocysts so all samples could be compared (Figure 1E).

Samples were hybridized on a microarray slide containing almost 44,000 probes per array coding for ~ 14,000 gene transcripts indicating that for a subset of genes more than one probe was present. If the position of the probe is nearer to the 3’ end of the corresponding gene, signal intensity is expected to be higher [25] and chance of incorrect signal by variations in RNA integrity is smaller [26]. Therefore, the expression of the probes corresponding to the most 3’ ends of genes was used for the analysis [27,28]. Gene expression levels in morula, blastocyst, ICM and TE were determined and a hierarchical clustering analysis was performed. The constructed heat map shows clear pairing of the morula, ICM and TE samples (Figure 1F). This is particularly important for the TE and ICM samples since these were manually dissected and confirms the reproducibility of the dissection. We used mechanical isolation of ICM from TE using tungsten needles. A selection of isolated ICMs was stained for CDX2 and GATA6 to identify the contribution of TE cells to the pooled microarray samples. The isolated ICMs contained only ~20% CDX2 positive TE (Figure 1G). Since however some TE cells remained attached to the ICM, throughout the manuscript “ICM” refers to the ICM containing few TE cells. In the TE samples all cells were CDX2 and GATA6 positive (Figure 1H). Blastocyst samples did not pair since their difference with the reference is minimal indicated by a near black appearance in the heat map. A principal component analysis (PCA) further identified four categories according to cell type or developmental stage. In a 2D plot, morula samples separate the farthest from the other samples. TE and ICM samples are clearly separated from each other with the blastocyst replicates in between (Figure 1I).

**Figure 2:**
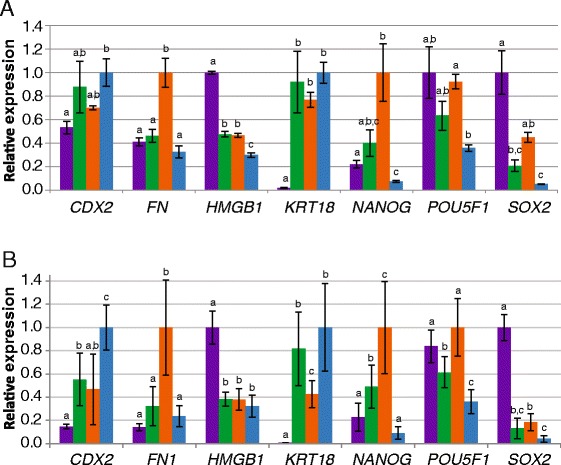
Relative gene expression. Microarray data were analysed for selected genes known for their expression in morula (purple), blastocyst (green), ICM (orange) and TE (blue) (**A**). The same genes were analysed by qRT-PCR in 4–6 samples per embryo stage or cell type revealing the same pattern (**B**). Normalization was performed with the reference genes encoding RPL15, SDHA and YWHAZ. Y-axis depicts relative mean expression to sample with highest expression set at 1 per gene. Bars with different letters are significantly different (p < 0.05) and error bars indicate standard deviation.

To further confirm the specificity of the samples, expression levels of genes that are known to be differentially expressed in bovine embryos were compared. Of the selected genes *HMGB1*, *SOX2* and *POU5F1* (coding for OCT4 protein) are known to be expressed at relatively high levels in bovine morula embryos [29-31], *CDX2* and *KRT18* have the highest expression levels in TE [30,32] while *FN1* and *NANOG* are abundantly expressed in the ICM [30]. The relative expression levels of the selected genes as determined by microarray analysis in morula, blastocyst, ICM and TE were as expected, with highest levels of *HMGB1* and *SOX2* expression in morulae, *POU5F1* expression predominantly in morulae and ICM, highest *FN1* and *NANOG* expression in the ICM and *CDX2* and *KRT18* expression at highest levels in TE (Figure 2A). These selected genes and five additional genes (Additional file 1: Figure S1) were also analysed for their expression levels by qRT-PCR, revealing a similar expression profile validating sample identity and demonstrating that the microarray data accurately reflect relative expression levels (Figure 2B).

### Genes differentially expressed between morulae and blastocysts

**Figure 3:**
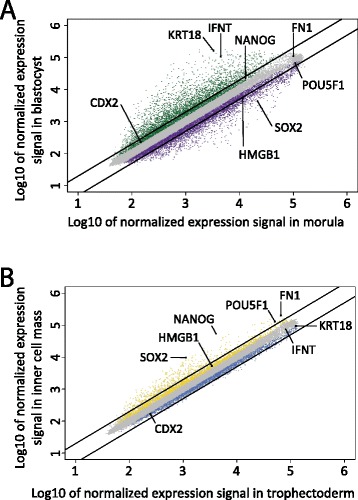
Relative gene expression in blastocyst versus morula and inner cell mass versus trophectoderm samples. All probes representing genes (grey) were plotted for their expression levels. Black lines represent the 2-fold cut off. Axes are Log10 transformed and depict the normalized expression signal in the indicated sample. Genes analysed in qRT-PCR are indicated. (**A**) Genes with significant (p ≤ 0.05) higher expression levels in blastocyst or morula are indicated with green or purple dots, respectively. (**B**) Genes with significant (p ≤ 0.05) higher expression levels in inner cell mass or trophectoderm are indicated with orange or blue dots, respectively.

Most differences in gene expression were found between morulae and blastocysts. When gene expression in morulae was compared with that in blastocysts using a >2-fold difference with p-value <0.05 as cut-off, 3,774 genes were differentially expressed. In the blastocyst, 1,960 genes were up-regulated, up to 107-fold, while 1,814 genes were expressed at higher levels in morulae (Figure 3A and Additional file 2: Table S1). The 25 most differentially expressed genes were expressed with at least a 30-fold difference of which only 1 gene (*ETNPPL*) was expressed at higher levels in the morula (Table 1).

**Table 1:** **List of the most differentially expressed genes between blastocyst and morula**

**Rank**	**Entrez Gene name**	**Gene discription**	**AgriGO ID**	**FC**
1	PLS1	Plastin-1	ENSBTAP00000053019	107.2
2	PAGE4	G antigen family C member 1	ENSBTAP00000047904	103.9
3	PGHS-2	prostaglandin-endoperoxide synthase 2 (prostaglandin G/H synthase and cyclooxygenase) (PTGS2), mRNA.	ENSBTAP00000018774	94.1
4	ANXA3	annexin A3	ENSBTAP00000042843	70.5
5	ALDH1A3	aldehyde dehydrogenase 1 family, member A3	ENSBTAP00000012030	64.9
6	SEPP1	selenoprotein P, plasma, 1 (SEPP1), mRNA	NP_776884	57.8
7	GCA	grancalcin, EF-hand calcium binding protein	ENSBTAP00000024550	54.1
8	MYOF	myoferlin	ENSBTAP00000038769	52.0
9	KRT8	keratin, type II cytoskeletal 8	ENSBTAP00000001108	51.9
10	KRT18	KRT18 proteinUncharacterized protein	ENSBTAP00000001988	49.2
11	SLC23A1	solute carrier family 23 (ascorbic acid transporter), member 1	ENSBTAP00000010823	48.7
12	ZFP37	Uncharacterized protein	ENSBTAP00000024598	45.6
13	PRSS22	Uncharacterized protein	ENSBTAP00000021967	43.2
14	AGRN	agrin	ENSBTAP00000017563	39.4
15	ETNPPL	Bos taurus alanine-glyoxylate aminotransferase 2-like 1 (AGXT2L1), mRNA.	ENSBTAP00000013587	−38.8
16	CYP51A1	Lanosterol 14-alpha demethylase	ENSBTAP00000002582	36.3
17	LDLR	low-density lipoprotein receptor	ENSBTAP00000016342	34.4
18	CNN2	Calponin-2	ENSBTAP00000027670	33.8
19	LOC787705	Uncharacterized protein		33.7
20	HMOX1	Heme oxygenase 1	ENSBTAP00000020701	33.2
21	PLAU	Urokinase-type plasminogen activatorUrokinase-type plasminogen activator long chain AUrokinase-type plasminogen activator short chain AUrokinase-type plasminogen activator chain B	ENSBTAP00000007806	32.9
22	PQLC3	PQ-loop repeat-containing protein 3	ENSBTAP00000027051	31.6
23	PDZK1	Na(+)/H(+) exchange regulatory cofactor NHE-RF3	ENSBTAP00000007638	31.0
24	LOC523509	Uncharacterized protein	ENSBTAP00000050990	30.9
25	TMEM20	Bos taurus solute carrier family 35, member G1 (SLC35G1), mRNA.	ENSBTAP00000045606	30.0

The 25 most differentially expressed genes (≥30-fold; p ≤ 0.05) between blastocyst and morula are listed. FC = fold-change and positive values indicate higher expression in blastocyst and negative values have higher expression in morula.

To identify properties of the differentially expressed genes a Parametric Analysis of Gene Set Enrichment (PAGE) was performed using AgriGO [33] and a list of the five most-enriched gene ontology (GO) terms per categorie was generated (Table 2). A large group of differentially expressed genes was involved in the *lipid metabolic process* (GO:0006629) in the blastocyst. A much smaller group of genes that function in *pepsin A activity* (GO:0004194) was over-represented in blastocysts and indeed, a large group of genes up-regulated in the blastocyst located to the *plasma membrane* (GO:0005886). Compared to the blastocyst, in the morula more genes were involved in the *nucleobase-containing compound metabolic process* (GO:0006139) and more specifically in *RNA processing* (GO:0006396). Morula up-regulated genes were found in the enriched molecular function GO terms *nucleic acid binding* (GO:0003676) and *transcription regulator activity* (GO:0030528).

**Table 2:** **AgriGO parametric analysis of gene set enrichment (PAGE) analysis (blastocyst versus morula)**

**GO_acc**	**Ontology**	**Description**	**Genes #**	**p-value**	**FDR**
GO:0006629	P	**lipid metabolic process**	311	0	0
GO:0016070	P	RNA metabolic process	840	2.70E-29	2.70E-26
GO:0006396	P	RNA processing	242	1.00E-28	7.00E-26
GO:0010467	P	gene expression	1068	6.50E-22	3.20E-19
GO:0006139	P	nucleobase, nucleoside, nucleotide and nucleic acid metabolic process	1169	4.20E-20	1.70E-17
GO:0003676	F	nucleic acid binding	929	5.70E-35	2.40E-32
GO:0003723	F	RNA binding	252	1.00E-20	2.20E-18
GO:0003677	F	DNA binding	623	7.00E-19	1.00E-16
GO:0030528	F	transcription regulator activity	445	4.40E-11	4.70E-09
GO:0004194	F	**pepsin A activity**	14	1.40E-10	1.20E-08
GO:0016020	C	**membrane**	1629	0	0
GO:0005886	C	**plasma membrane**	907	0	0
GO:0044425	C	**membrane part**	1280	0	0
GO:0044421	C	**extracellular region part**	564	0	0
GO:0016021	C	**integral to membrane**	1012	0	0

Genes differentially expressed between blastocyst and morula were assessed for their properties described by gene ontology (GO) terms in respect to their relative expression. The five most significantly enriched GO-terms (according to p-value) are listed for each GO domain. P = Biological Process, F = Molecular Function and C = Cellular Component. Bold descriptions indicate enrichment for blastocyst. Regular descriptions indicate enrichment for morula. FDR = False Discovery Rate (Hochberg).

### Genes differentially expressed between ICM and TE

When gene expression in the ICM was compared with that of the TE, 497 genes were differentially expressed. Here, the majority (406) of the differentially expressed genes were expressed at higher levels in the ICM. Of all genes, the difference in expression levels of *NANOG* was the largest (13-fold up-regulated in the ICM, Table 3 and Additional file 2: Table S2). *SOX2* and *POU5F1* that together with *NANOG* form the core transcriptional regulatory circuitry in pluripotent cells were also over-expressed in the ICM compared with TE (Figure 3B).

**Table 3:** **List of the most differentially expressed genes between inner cell mass and trophectoderm**

**Rank**	**Entrez gene name**	**Gene description**	**AgriGO ID**	**FC**
1	NANOG	Homeobox protein NANOG	ENSBTAP00000027863	13.2
2	UPP1	uridine phosphorylase 1	ENSBTAP00000011088	8.9
3	SOX2	SRY (sex determining region Y)-box 2	ENSBTAP00000015411	8.6
4	CAV1	Caveolin-1	ENSBTAP00000023751	7.8
5	AK3L1	Adenylate kinase isoenzyme 4, mitochondrial	Q0VCP1	7.5
6	LOC616039	pancreatic trypsin inhibitor-like	XP_873093	7.4
7	OTX2	agilent:“Bos taurus orthodenticle homeobox 2 (OTX2), mRNA	ENSBTAP00000019616	7.2
8	GPC4	agilent:“Bos taurus glypican 4 (GPC4), mRNA	ENSBTAP00000027510	7.1
9	HAS2	hyaluronan synthase 2	ENSBTAP00000026503	7.1
10	SLC4A7	solute carrier family 4, sodium bicarbonate cotransporter, member 7	AAI42306.1	7.1
11	HNF4A	agilent:“Bos taurus hepatocyte nuclear factor 4, alpha (HNF4A), mRNA	ENSBTAP00000016078	7.0
12	CLIC6	CLIC6 chloride intracellular channel 6	ENSBTAP00000002299	6.9
13	LGALS4	galectin-4	ENSBTAP00000021701	6.8
14	ID1	DNA-binding protein inhibitor ID-1	ENSBTAP00000021521	6.7
15	FLRT3	fibronectin leucine rich transmembrane protein 3	ENSBTAP00000004298	6.6
16	PDGFRA	agilent:“Bos taurus platelet-derived growth factor receptor, alpha polypeptide (PDGFRA), mRNA	ENSBTAP00000009441	6.6
17	NID1	nidogen 1	ENSBTAP00000009531	6.5
18	KIT	Mast/stem cell growth factor receptor	ENSBTAP00000003498	6.5
19	GRP	agilent:“Bos taurus gastrin-releasing peptide GRP mRNA, complete cds.	ENSBTAP00000006297	6.5
20	LOC100139916	LOC100139916 interleukin 32-like		6.5
21	TKTL1	Transketolase-like protein 1	ENSBTAP00000036249	6.4
22	A2M	Alpha-2-macroglobulin	ENSBTAP00000006167	6.4
23	PDYN	proenkephalin-B preproprotein	AAI51344.1	6.1
24	ACTG2	actin, gamma 2, smooth muscle, enteric	ENSBTAP00000036954	6.0
25	MME	membrane metallo-endopeptidase	ENSBTAP00000002681	6.0

The 25 most differentially expressed genes (>6 fold; p ≤ 0.05) between inner cell mass and trophectoderm are listed according to their relative expression. FC = Fold change and positive values indicate higher expression in inner cell mass.

**Figure 4:**
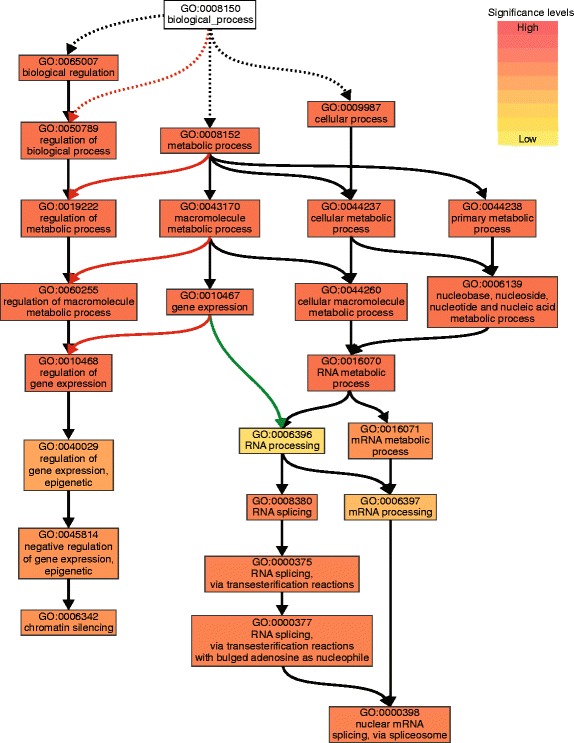
Biological processes in inner cell mass (partial). All genes expressed at higher levels in inner cell mass compared with trophectoderm were assessed for their enrichment (GO term analysis in AgriGO) in biological processes (GO:0008150). The hierarchical tree graph contains a highly enriched group of processes ending in *chromatin silencing* (GO:0006342) and *nuclear mRNA splicing, via spliceosome* (GO:0000398).

For human ES cells, a network of NANOG-, SOX2- and POU5F1-target genes that encode transcription factors and chromatin modulators has been established [34]. From the list of positively regulated genes in human ES cells, apart from the core network only expression of *STAT3* and *ZIC3* was significantly up-regulated in bovine ICM versus TE (Additional file 2: Table S2). Again, we performed a parametric analysis of gene set enrichment with the genes differentially expressed between ICM and TE in AgriGO [33]. With a false discovery rate (FDR) ≤0.1, *enzyme linked receptor protein signaling pathway* (GO:0007167) and *peptidyl-tyrosine phosphorylation* (GO:0018108) terms were enriched for the ICM whereas for the TE, genes were enriched in the *sterol biosynthetic process* (GO:0016126) (Table 4). Only genes up-regulated in respect to the TE were used for a singular enrichment analysis (SEA) in order to identify characteristics specific for the cells of the ICM (Additional file 2: Table S3). In particular, terms containing genes involved in *RNA splicing* (GO:0008380) and *regulation of gene expression* (GO:0010468) were enriched even to a more specific level of enriched child terms like *nuclear mRNA splicing* (GO:0000398) and *chromatin silencing* (GO:0006342), respectively (Figure 4, Additional file 2: Table S3 and Additional file 3: Figure S2).

**Table 4:** **AgriGO parametric analysis of gene set enrichment (PAGE) analysis (inner cell mass versus trophectoderm)**

**GO_acc**	**Ontology**	**Description**	**Genes #**	**p-value**	**FDR**
GO:0016126	P	sterol biosynthetic process	12	1.40E-11	9.10E-09
GO:0016125	P	sterol metabolic process	20	2.00E-09	6.50E-07
GO:0008202	P	steroid metabolic process	28	1.00E-07	1.70E-05
GO:0008203	P	cholesterol metabolic process	18	8.90E-08	1.70E-05
GO:0006694	P	steroid biosynthetic process	20	2.00E-07	2.60E-05
GO:0006629	P	lipid metabolic process	62	6.40E-07	6.10E-05
GO:0008610	P	lipid biosynthetic process	34	6.40E-07	6.10E-05
GO:0006720	P	isoprenoid metabolic process	13	2.10E-05	1.70E-03
GO:0006066	P	alcohol metabolic process	54	1.50E-04	1.10E-02
GO:0044255	P	cellular lipid metabolic process	39	2.20E-04	1.40E-02
GO:0010038	P	response to metal ion	14	3.70E-04	2.20E-02
GO:0006721	P	terpenoid metabolic process	11	5.20E-04	2.90E-02
GO:0023034	P	**intracellular signaling pathway**	65	1.70E-03	8.70E-02
GO:0018212	P	**peptidyl-tyrosine modification**	12	2.30E-03	9.40E-02
GO:0018108	P	**peptidyl-tyrosine phosphorylation**	12	2.30E-03	9.40E-02
GO:0007167	P	**enzyme linked receptor protein signaling pathway**	39	2.00E-03	9.40E-02
GO:0031090	C	organelle membrane	70	1.90E-04	7.40E-03
GO:0005773	C	vacuole	18	1.40E-04	7.40E-03
GO:0042175	C	nuclear envelope-endoplasmic reticulum network	23	1.40E-04	7.40E-03
GO:0005789	C	endoplasmic reticulum membrane	22	4.70E-04	1.00E-02
GO:0005764	C	lysosome	17	5.10E-04	1.00E-02
GO:0000323	C	lytic vacuole	17	5.10E-04	1.00E-02
GO:0005768	C	endosome	23	1.40E-03	2.20E-02
GO:0012505	C	endomembrane system	62	1.50E-03	2.20E-02
GO:0044432	C	endoplasmic reticulum part	25	2.30E-03	3.00E-02

All genes differentially expressed between ICM and TE were assessed for their properties described by gene ontology (GO) terms in respect to their relative expression. This reveals 25 enriched GO-terms with a FDR ≤ 0.1 in the GO domains Biological Process (P) and Cellular Component (C). Ranked according to z-score. Bold descriptions indicate enrichment for inner cell mass. Regular descriptions indicate enrichment for trophectoderm. FDR = False Discovery Rate (Hochberg).

### Genes differentially expressed between MAPK-inhibited and control ICMs

**Figure 5:**
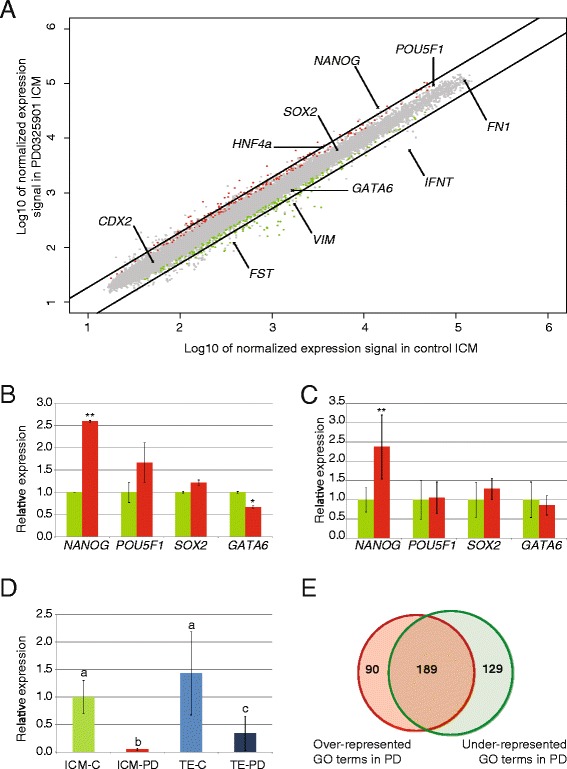
Expression in and GO-analysis of MAPK-inhibited and control inner cell mass. All probes representing genes (grey) were plotted for their expression levels (**A**). Black lines represent the 2-fold cut-off. Axes are Log10 transformed and depict the normalized expression signal in the indicated sample. Genes discussed in text (and analysed by qRT-PCR) are indicated. Genes with significant (p ≤ 0.05) higher expression levels in MAPK-inhibited and control inner cell masses are indicated with red and green dots, respectively. MAPK-inhibited ICM (red) gene expression of *NANOG*, *POU5F1*, *SOX2* and *GATA6* were determined by microarray (**B**) and qRT-PCR (**C**) presented relative to gene expression in control ICM (green). Significant differences are indicated; * p < 0.05; ** p < 0.005. *IFNT* expression (**D**) was determined by qRT-PCR in control TE (light blue), PD0325901-treated TE (dark blue) and PD0325901-treated ICM (red) and presented relative to control ICM (green). Normalization was performed with the reference genes *RPL15*, *SDHA* and *YWHAZ*. Error bars indicate standard deviation and data bars with different letters are significantly different (p < 0.05). A Venn diagram (**E**) shows the numbers of overrepresented (red; total 279) and underrepresented (green; 318 in total) GO-terms in the MAPK-inhibited inner cell mass. 189 GO-terms are in common.

It has been described that culture of ICM cells in the presence of an FGFR inhibitor, a MAPK inhibitor and an inhibitor of GSK3β, the so-called 3i culture system, leads to stable ES cell lines from non-permissive mouse strains [10,35] and rat embryos [20]. Culture of mouse embryos in the presence of a MAPK inhibitor resulted in all cells of the ICM expressing *Nanog* while the expression of *Gata6* was down-regulated [11]. Similarly, when bovine embryos were cultured in the presence of the MAPK inhibitor PD0325901, a larger percentage, although not all, of the ICM cells expressed NANOG [13]. Therefore, in order to identify NANOG target genes and genes that may be important for pluripotency, ICMs were isolated from bovine embryos cultured in the presence of the MAPK inhibitor PD0325901. Gene expression in these ICMs was compared with that from control ICMs using microarray analysis. In total 94 genes were differentially regulated between control (DMSO) and MAPK-inhibited ICMs, ≥2-fold difference with p-value ≤ 0.05 as cut-off, with the expression of 44 genes up-regulated and the expression of 50 genes down-regulated (Additional file 2: Table S4). As expected, *NANOG* expression was up-regulated in the MAPK-inhibited ICM as detected by microarray analysis (Figure 5A,B). Expression differences between control ICM and ICM from embryos exposed to the MAPK inhibitor detected by qRT-PCR verified the microarray data (Figure 5B,C and Additional file 4: Figure S3). Furthermore, immunostaining showed an increase in the percentage of NANOG expressing cells in the ICM after MAPK inhibition (Additional file 5: Figure S4) as we had previously established [13]. To our surprise several interferon coding genes were dramatically down-regulated after MAPK inhibition such as *IFNW1* and *IFNT* (Figure 5A). Gene expression analysis by qRT-PCR confirmed the microarray results and further showed a decreased *IFNT* expression in TE and to a greater extend in ICM upon MAPK inhibition (Figure 5D).

Genes up-regulated in the PD-treated or in the control ICMs were enriched in 189 of the same GO terms after a singular enrichment analysis containing at least two genes and a FDR ≤0.1 (Additional file 2: Table S5 and Figure 5E). When the relative gene expression was taken into account only 6 GO terms were found to be enriched with a FDR < 0.1, of which 4 were under-represented in the PD-treated ICMs (Table 5). The only function-describing term was *receptor binding* (GO:0005102) and this term was under-represented in the PD group.

**Table 5:** **AgriGO parametric analysis of gene set enrichment (PAGE) analysis (MAPK inhibited versus control)**

**GO_acc**	**Ontology**	**Description**	**Genes #**	**p-value**	**FDR**
GO:0005102	F	receptor binding	12	0.0016	0.027
GO:0005615	C	extracellular space	26	7.60E-05	0.003
GO:0044421	C	extracellular region part	28	0.00019	0.0036
GO:0005576	C	extracellular region	34	0.0033	0.043
GO:0043232	C	**intracellular non-membrane-bounded organelle**	18	0.0093	0.073
GO:0043228	C	**non-membrane-bounded organelle**	18	0.0093	0.073

All genes differentially expressed between MAPK-inhibited and control ICMs were assessed for their properties described by gene ontology (GO) terms in respect to their relative expression. This revealed 6 enriched GO-terms with a FDR ≤ 0.1 in the GO domains Molecular Function (F) and Cellular Component (C). Bold descriptions indicate enrichment in MAPK inhibited ICM. Regular descriptions indicate enrichment in control ICM.

### Cross-comparisons

**Figure 6:**
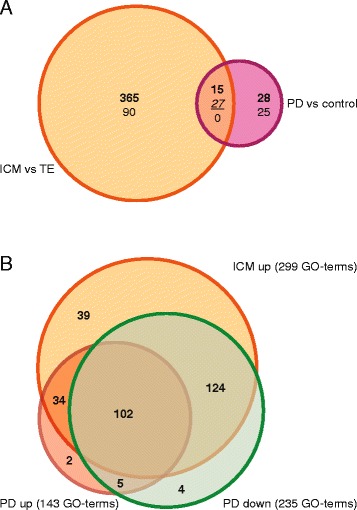
Gene expression and processes in the inner cell mass. (**A**) Of the 497 genes differentially expressed (≥2-fold; p ≤ 0.05) between ICM and TE (orange) several are up-regulated (**bold**) or down-regulated (regular) and 42 of these are also differentially expressed when inner cell masses treated with the MAPK-inhibitor PD0325901 (PD) are compared with control inner cell masses (magenta). This latter comparison retains 95 differentially expressed genes. Of the 42 shared genes 15 are up-regulated in both comparisons and 27 are contra-regulated (italics and underlined). (**B**) A gene ontology analysis with 41 ICM up-regulated genes (orange), 16 PD up-regulated (red) and 26 PD down-regulated (green) genes indicates several GO-terms of which 34 are enriched in both up-regulated gene analyses.

The 497 genes differentially expressed in the ICM versus TE and the 95 differentially expressed genes in the MAPK inhibited versus control ICMs were compared revealing that 42 genes were shared (Table 6). Of these genes 15 were both up-regulated in ICM and after MAPK inhibition while 27 were contra-regulated (Figure 6A). Only *PRPH* was expressed at lower levels in ICM than in TE and 26 genes were down-regulated after MAPK inhibition and the expression of 16 genes was further up-regulated (Table 6). Although it has been described that NANOG activates *POU5F1* and *SOX2* transcription, expression of these genes was not significantly altered after up-regulation of *NANOG* expression by MAPK inhibition.

**Table 6:** **List of genes differentially expressed in ICM versus TE and PD treated versus control ICMs**

**Gene name**	**FC (ICMvsTE)**	**FC (PDvsControl)**
ADH6	3.336	−2.993
AHCYL2	5.379	2.068
C8A	2.558	2.009
CD8B	3.392	−2.010
CKB	3.660	2.062
CTSC	3.064	−2.517
CYP1A1	4.041	−2.563
DHRS7	3.717	2.170
DUSP4	3.685	−6.320
EMILIN2	5.463	2.697
FBLN1	2.215	−2.160
FST	2.468	−3.235
GKN2	2.615	−2.426
HAS2	7.117	−2.237
JAM2	2.205	−2.186
LOC100139049	2.661	2.276
LOC100139916	6.461	−2.002
LOC100140174	3.096	2.880
LOC616039	7.437	−2.203
MAP1B	3.657	2.032
MEIS2	3.638	−2.508
MFAP5	4.520	−2.403
MYL9	2.257	3.305
NANOG	13.215	2.575
NID1	6.536	−2.072
P4HA3	3.279	2.083
PDGFRA	6.583	−2.116
PDYN	6.053	−2.398
PHLDA1	2.679	−2.133
PRPH	−2.552	2.124
PRSS12	4.640	2.334
RSPO3	2.572	−2.619
S100B	3.875	3.516
SELP	2.758	2.466
SERPINA5	5.908	−7.057
SERPINH1	2.673	−2.110
SLC1A3	3.230	−2.249
SPRY4	4.314	−2.065
TGM2	3.363	−2.231
TIFA	3.394	−2.569
unknown	2.868	2.076
VIM	4.636	−2.827

Alphabetical ranking of differentially expressed genes (p < 0.05) in inner cell mass versus trophectoderm (ICMvsTE) and MAPK-inhibited versus control inner cell masses (PDvsControl). FC = Fold change in respect to ICM or PD.

The expression of *GATA6* was significantly down-regulated in the ICM after MAPK inhibition, although the difference was less than 2-fold and was not significant in the qRT-PCR analysis. The expression of 26 other ICM-specific genes was significantly down-regulated upon MAPK inhibition suggesting that these genes are involved in primitive endoderm formation (Figure 5 and Table 6). Expression of a number of the genes was also analysed by qRT-PCR showing a similar pattern and validating the microarray data (Figure 5 and Additional file 4: Figure S3). Indeed mouse follistatin, coded by *Fst*, has been implicated as a marker for primitive endoderm derivatives [36]. Other genes down-regulated in the MAPK inhibited bovine ICMs themselves code for negative regulators of MAPK activity such as *DUSP4* and *SPRY4*.

The 42 shared genes were analyzed for their properties by gene ontology analysis. A PAGE analysis in AgriGO revealed 59 enriched GO-terms of which none had a FDR < 0.1. When the SEA was performed using either ICM up-regulated, PD up-regulated or PD down-regulated genes (Additional file 2: Table S6), a Venn diagram with the enriched terms (FDR < 0.1) revealed 34 enriched GO-terms shared between the up-regulated gene comparisons (Figure 6B, Additional file 2: Table S6).

## Discussion

During bovine pre-implantation development several cell types display a pluripotent character. The failure in generating true pluripotent ES cell lines from *Bos taurus* embryos however indicates that, compared with murine and human embryos, other genes are involved in maintenance of pluripotency, that the correct embryonic stage with pluripotent cells has not been used, or that the culture conditions employed did not sufficiently inhibit differentiation. Interestingly, induced pluripotent stem (iPS) cells generated from bovine cells also behave differently than mouse iPS cells. Similar to porcine iPS cells, the introduced transgenes are not silenced in the currently used culture conditions but remain expressed in these cells suggesting that other factors are needed for maintenance of pluripotency [37]. We performed a microarray analysis comparing morula, blastocyst, ICM and TE gene expression profiles to identify genes possibly involved in pluripotency. Further enrichment of the pluripotent character of the ICM was achieved by inhibiting the MAPK pathway through exposure to the MEK inhibitor PD0325901 during in vitro culture thereby increasing the percentage of *NANOG* expressing cells in the ICM/epiblast [13,14].

To obtain samples for the microarray analysis embryos were cultured up to the morula stage or blastocyst stage. Blastocysts were dissected manually to separate ICM and TE. The advantage of this technique is that ICM and TE are isolated from the same embryo, in contrast to for example a technique like immunosurgery. Manually dissecting blastocysts is challenging however and it is unavoidable that few TE cells remain attached to the ICM. We therefore verified that the separation of the two cell types was successful. For separation of ICM from TE, Nagatomo et al. have used either a micromanipulator or mild trypsin treatment to separate ICM [38]. ICMs isolated using the micromanipulator still contained 43.3% TE cells [38]. In our hands the percentage of TE cells remaining in the ICM isolates was ~20% as determined by CDX2 expression, indicating a low contribution of TE cells to the ICM transcriptome. The disadvantage of mild trypsin treatment to isolate ‘pure’ ICM cells, is that TE and ICM cannot be compared from the same embryo and that the trypsin treatment by itself may cause a difference in gene expression. The observation that duplicate samples paired together and that TE and ICM clustered apart from each other together with the expected expression patterns of known TE- and ICM-specific genes in the microarray as well as by qRT-PCR indicates that indeed the separation was specific and reproducible.

Several genes were represented on the array by multiple probes and in those cases we only used the expression data of the most 3’-located probe. Unfortunately, *the Bos taurus* genome is not completely annotated [39] and indeed approximately 5% of the probes representing genes differentially expressed between blastocyst and morula could not be identified. The other comparisons could be made with all probes linked to a known differentially expressed gene. For the Gene Ontology analysis genes need to be associated with a GO-term. Not all genes are associated with a GO-term and therefore 3.7% – 5.3% of the genes could not be analysed in the AgriGO gene ontology analysis.

In vitro derived embryos were used as this enabled us to generate the numbers needed for RNA extraction. Particularly for the ICM and TE samples large numbers of embryos were needed to obtain sufficient amounts of RNA for hybridization (Additional file 2: Table S7). Although a significant difference in gene expression between in vitro and in vivo derived embryos has been demonstrated [40] the birth of healthy animals from in vitro derived embryos indicates that the pathways for pluripotency are functional in these embryos. When gene expression was compared between different stages of in vivo derived bovine embryos most genes were found to be differentially expressed between early development (oocyte-4 cell stage) and later stages (8-cell stage-blastocyst) [40]. Most likely these differences in gene expression are caused by embryonic genome activation around the 8-cell stage [40-42]. A larger number of genes (~1800) was expressed in in vivo derived oocytes compared with in vitro matured oocytes [40], indicating that in our study with in vitro derived embryos important genes may not have been detected. However, since in vitro derived embryos are commonly used for embryo transfer and give rise to healthy animals, it can be expected that genes important for pluripotency are sufficiently expressed and the pathways for pluripotency are functional in in vitro derived embryos.

We started our analysis by comparing gene expression in blastocysts with that in morulae. This indicated that most differentially expressed genes are expressed at higher levels in the blastocyst but a GO-analysis revealed that most genes expressed at higher levels in morulae are involved in gene transcription. This might be a result of the embryonic genome activation initiated during the 8-16 cell stage in cattle embryos preceding the morula stage [40-42]. Next we tried to identify genes involved in pluripotency by comparing gene expressions in ICM and TE. Mouse and human ES cell pluripotency is regulated by NANOG, SOX2 and OCT4, and these factors enhance each other’s transcription [43-45]. Indeed, their gene expression levels were found higher in the bovine ICM samples compared with TE. Remarkably, in the comparison of the ICM with the TE, expression of genes in the GO-category *RNA splicing* was specifically up-regulated in the ICM. This indicates a higher transcriptional activity in ICM cells than in TE cells. This is further reflected in the >400 genes up-regulated in ICM compared with TE. Using deep sequencing, Ozawa et al. examined genes differentially expressed between ICM and TE of day 8 in vitro derived embryos [32]. All of 8 ICM-characteristic genes that Ozawa et al. found were also up-regulated in our study, except for *ZC3HAV1* and *Il6R*. Expression of *Il6R* was indeed significantly up-regulated in the ICM but the difference was below the cut-off used (2-fold). These results confirm the specificity and reliability of the ICM isolation and microarray analysis. Compared with our results Ozawa et al. found more genes (870 versus 497 in our study) to be differentially expressed between ICM and TE, most likely because of the less stringent cut-off value used (1.5 versus 2.0 fold difference in our study) [32].

By enhancing the overall *NANOG* expression in the ICM we had anticipated that *SOX2* and *POU5F1* expression were similarly enhanced. Surprisingly however, in ICMs from embryos cultured in the presence of a MAPK inhibitor, gene expression levels of *NANOG* were up-regulated while those of *POU5F1* and *SOX2* remained relatively unchanged*.* These results suggest that in bovine cells *NANOG* by itself is not sufficient in maintaining the core pluripotency network.

An unexpected result was the expression of several interferon-coding genes in the ICM. Various reports have described exclusive *IFNT* expression in trophectoderm or TE derived cell lines [46-48]. We detected *IFNT* expression in the isolated ICMs at similar levels as in TE however and the expression in the ICM was down-regulated upon MAPK inhibition even to a greater extent than in TE. In ungulates, interferon tau (coded by *IFNT*) expression by TE is important for maternal pregnancy recognition [49]*.* In bovine day 7 blastocysts interferon tau has been detected at varying intensity in the TE and was concentrated at the border of the ICM and TE [48]. By dissecting the ICM, part if not all of the interferon tau-positive adjacent cells have been included in the ICM samples accounting for the observed *IFNT* expression in the ICM samples. Together with *CDX2* predominantly expressed in TE cells and capable of increasing *IFNT* transcription [46,50], this might explain the greater expression reduction found in ICM (20-fold) than in TE (4-fold). Nevertheless, our results and the previously reported location of interferon tau expression [48] do not exclude *IFNT* expression in the ICM even though its function in the ICM is unknown.

After exposure to PD0325901 expression of *NANOG* in the ICM was enhanced as compared to control ICMs. In the mouse more than 3,000 genes have been identified containing NANOG binding sites [51]*.* Of the 42 genes identified to be differentially expressed in bovine ICMs and after MAPK inhibition, only five were homologous to murine genes containing NANOG binding sites. Of those genes only expression of *NANOG* was up-regulated after MAPK inhibition. Of the remaining four*, CD8B, DUSP4, JAM2* and *SPRY4,* the expression was enhanced in the ICM but their expression was down-regulated after MAPK inhibition. The role of the glycoprotein CD8B in early embryonic development, and more specifically in the ICM, is unclear. Its expression can be regulated however by MAPK signalling [52,53] possibly accounting for the observed down-regulation after PD0325901 treatment. DUSP4 is suggested to function in the negative feedback control of MAPK signalling specifically dephosphorylating ERK1/2 [54,55]. Also SPRY4 is known for its involvement in the MAPK pathway by interacting with GRB2 and GAP1 and as such inhibiting RAS activation [56] and antagonizing FGF activity [57]. Therefore, the down regulation of *DUSP4* and *SPRY4* expression by MAPK inhibition is most likely a direct result of the MAPK inhibition rather than result from the up-regulation of *NANOG* expression*. JAM2* is expressed in both embryonic and adult stem cell lines [58] and its expression is enhanced in undifferentiated mouse ES cells compared to early stages of differentiation. Since mouse ES cells that genetically lack *Jam2* maintain pluripotency however, the function of JAM2 in stem cells remains unknown [59]. In mouse Sertoli cells inhibition of ERK activity did not affect *Jam2* transcription [60], suggesting that the observed reduced *JAM2* expression resulted from increased *NANOG* levels*.* Interestingly*, JAM2* expression was also down-regulated after OCT4 had been exogenously introduced into human cells, suggesting that low levels of JAM2 induce or indicate differentiation [61]. In our bovine ICMs *POU5F1* expression was however not significantly up-regulated after enhanced *NANOG* expression. Surprisingly, no other genes that had been identified as overlapping NANOG putative targets in mouse and human ES cells [51] appeared to be up- or down-regulated in bovine ICMs with enhanced *NANOG* expression*.*

Apart from the core pluripotency markers NANOG, SOX2 and OCT4, other transcription factors are reported to be involved in mouse or human pluripotency. Of all transcription factors differentially expressed between ICM and TE, *OTX2* ranked third and was 7.2-fold higher expressed in ICM. In mouse ES cells OTX2 was reported to be required for the transition to a stable epiblast stem cell condition [62]. Recently, it was shown that OTX2 is one of the earliest transcription factors to be activated during exit from a naïve ground state in mES cells [63]. Although the MAPK pathway is important in cell differentiation [35] and therefore might influence *OTX2* expression we did not detect a difference in *OTX2* expression in the PD treated bovine ICMs. Together, these findings suggest that the ICMs under investigation were already in a “primed” state.

Transcription factors involved in the LIF or BMP pathway were also amongst the genes with up-regulated expression levels in the ICM. Although *BMP4* was not differentially expressed, *STAT3* (2.5-fold), *ID3* (2.7-fold) and *ID1* (6.7-fold) were expressed at higher levels in ICM than in TE. STAT3 is capable of suppressing mesoderm and endoderm commitment whereas ID genes suppress neuroectoderm commitment in mES cells. Fibronectin, with expression levels almost 4-fold higher in ICM, can induce *Id* expression and also NANOG is capable of activating STAT3 and inducing ID genes [64]. Up-regulated *NANOG* expression did however not induce *STAT3* or *ID* expression in MAPK inhibited ICMs. Although the level of expression might not be high enough, the increased expression of *STAT3*, *ID1* and *ID3* suggests that, although in a primed state, differentiation is not initiated yet in the bovine day 9 ICMs.

The transcription factor PRDM14 is implicated to act as a safeguard for maintaining pluripotency [65] and is uniquely expressed in mouse compacted morula, ICM, the early epiblast, primordial germ cells and ES cells [66-68]. Indeed, the expression of *PRDM14* was found to be up-regulated in bovine morulae compared to blastocysts (2.4-fold; p = 0.00097) and ICM versus TE (2.5-fold; p = 0.0015). It has been reported that in mouse ES cells PRDM14 attenuates FGF-induced differentiation [68]. We did however not observe a difference in levels of *PRDM14* expression in MAPK-inhibited ICMs (1.07 fold difference; p = 0.87), suggesting that the FGF- or MAPK-signalling pathways do not repress *PRDM14* expression in bovine pluripotent cells. Expression of the ZIC gene family members *ZIC2* and *ZIC3* was also up-regulated in the ICM (4.3-fold and 2.2-fold, respectively). *Zic2* and its orthologues are expressed in frog [69] and zebrafish [70], pregastrulation embryos and in mouse E0.5 and ICM of E4.5 embryos [71]. *Zic3* is implicated to play an important role in maintaining pluripotency in mouse ES cells [72] and contains OCT4, NANOG and SOX2 binding sites [34,51]. Indeed, after increased *NANOG* expression by MAPK inhibition *ZIC3* expression increases 1.5 fold (p = 0.038) but *ZIC2* expression decreased 2.2-fold (p > 0.05). Other reported genes to safeguard pluripotency such as *PARP1* and *PARP7* [73] were expressed at higher levels in morula than in blastocyst (*PARP7*), did not show a differential expression between ICM and TE and were not differentially expressed after MAPK inhibition. All together, these findings indicate that, despite the increased *ZIC3* and *NANOG* expression, MAPK inhibition by PD0325901 is insufficient to maintain a pluripotent state in bovine ICM cells.

## Conclusion

We have identified whole genome expression profiles of different stages of bovine embryos and TE and the pluripotent ICM of blastocysts. In addition, the transcriptome of ICMs with enhanced *NANOG* expression after inhibition of MAPK activity was established. Unfortunately, these expression profiles did not lead to (new) pathways or indications how to maintain pluripotency and possibly generate genuine bovine ES cells. Furthermore, it became apparent that although MAPK inhibition increased *NANOG* and *ZIC3* expression, this is insufficient to maintain pluripotency. Comparing transcription factor expression in the bovine ICMs used in the microarray with known expressions in mouse pluripotent cells indicates a “primed” or epiblast state. Therefore, the data presented in this paper can act as a starting point for further research on bovine pluripotency.

## Methods

### Bovine in vitro embryo culture and mechanical separation

Bovine embryo culture was performed at 39°C in a humidified atmosphere with 5% CO_2_, unless stated otherwise. Three to eight mm follicles of ovaries, obtained from a local slaughterhouse, were aspirated to retrieve COCs. Groups of 35–60 COCs were incubated for 23 hrs in 500 μl M199 (Life Technologies, Bleiswijk, The Netherlands) supplemented with 0.05 IU/ml recombinant hFSH (Organon, Oss, The Netherlands) and with 1% (v/v) penicillin-streptomycin (Life Technologies). Fertilization was performed as described previously [74] with modifications as described [75]. In short, matured COCs were transferred to fertilization medium (Fert-TALP) supplemented with heparin at a final concentration of 10 μg/ml (Sigma-Aldrich, Zwijndrecht, The Netherlands), 20 μM D-penicillamine (Sigma-Aldrich), 10 μM hypotaurine (Sigma-Aldrich), and 1 μM epinephrine (Sigma-Aldrich). Frozen-thawed sperm from a bull with proven fertility was centrifuged over a Percoll-gradient (GE Healthcare Europe GmbH, Eindhoven, The Netherlands) and added to the COCs at a final concentration of 1.0 × 10^6^ spermatozoa/ml. Fertilization day was considered as day 0. After incubation for 20 hrs the COCs were denuded by vortexing for 3 min and the cumulus-free oocytes were placed in synthetic oviduct fluid (SOF) medium. The presumptive zygotes were incubated at 39°C in a humidified atmosphere with 7% O_2_ and 5% CO_2_. At day 5 either morulae were collected or embryos were transferred to fresh SOF medium, cultured to blastocyst stage embryos and collected on day 9. Embryos were cultured until day 9 of development as this resulted in a higher percentage of hatching and hatched blastocysts [30,76,77], which facilitated ICM from TE separation. To ensure good quality embryos only stage code 7–9 blastocysts with quality code 1 or 2, according to the IETS manual, were collected [78,79]. To obtain ICMs containing a higher percentage of NANOG-expressing cells, SOF medium was supplemented with a final concentration of 0.5 μM PD0325901 (Stemgent, Cambridge, MA, USA) at day 5. Embryo culture for control ICM samples was performed with equal concentrations of the solvent DMSO.

Blastocysts collected to obtain inner cell mass and trophectoderm were placed in wash buffer containing 6.67 mg/ml NaCl (Merck, Schiphol-Rijk, The Netherlands), 0.24 mg/ml KCl (Merck), 0.168 mg/ml NaHCO_3_ (Sigma-Aldrich), 0.047 mg/ml NaH_2_PO_4_ (Merck), 0.217% (v/v) of a 60% sodium lactate solution (Sigma-Aldrich), 2.38 mg/ml HEPES (Sigma-Aldrich), 0.2% (v/v) phenolred (Sigma-Aldrich), 0.39 mg/ml CaCl_2_ · 2H_2_O (Sigma-Aldrich), 0.10 mg/ml MgCl_2_ · 6H_2_O (Merck), 0.11 mg/ml sodium pyruvate, 100U/ml Penicillin-Streptomycin (Life Technologies) and 6.0 mg/ml bovine serum albumin fraction 5 (MP Biomedicals, Santa Ana, CA, USA), set at an osmolality of 280 osmol/kg and adjusted to pH 7.3.

Sharpened tungsten needles were used to manually separate the trophectoderm from the ICM. This procedure was performed in wash medium under a stereo microscope.

### RNA isolation

Collected cells and embryos were harvested per tissue type or treatment and stored in 100 μl extraction buffer (Life Technologies) at −80°C until RNA isolation. RNA isolation and on column DNA digestion (Qiagen, Venlo, The Netherlands) was performed using the PicoPure® RNA isolation kit (Life Technologies) according to the manufacturer’s protocol. Total RNA quality and quantity assessment was performed by micro-electrophoresis on a Bioanalyzer 2100 using the RNA 6000 Pico LabChip kit (Agilent Technologies, Amstelveen, The Netherlands) according to manufacturer’s instructions. RNA was stored at −80°C until further use.

### Microarray gene expression analysis

Selected total RNA samples were compared in a common reference experiment design using 12 dual channel microarrays (8 for the stage-/cell-specific microarray and 4 for the ERK-inhibition microarray) with each sample hybridized against an identical common reference total RNA sample consisting of a pool of blastocysts total RNA. Within each group of two microarrays for each stage/tissue type/treatment, sample versus common reference hybridizations were performed in balanced dye-swap.

Microarrays used were bovine whole genome gene expression microarrays V2 (Agilent Technologies) representing 43,653 *Bos taurus* 60-mer oligos in a 4x44K layout.

cDNA synthesis, cRNA double amplification, labelling, quantification, quality control and fragmentation were performed with an automated system (Caliper Life Sciences NV/SA, Teralfene, Belgium), starting with 10–20 ng total RNA from each sample, all as previously described in detail [80,81]. Microarray hybridization and washing was with an HS4800PRO system with QuadChambers (Tecan, Mechelen, Belgie) using 700 ng, 1-2% Cy5/Cy3 labelled cRNA per channel as described [80]. Slides were scanned on an Agilent G2565BA scanner at 100% laser power, 30% PMT. After automated data extraction using Imagene 8.0 (BioDiscovery, Hawthorne, CA, USA), Loess normalization was performed [82] on mean spot-intensities. Gene-specific dye bias was corrected by a within-set estimate [83]. Data were further analysed by MAANOVA [84], modelling sample, array and dye effects in a fixed effect analysis. P-values were determined by a permutation F2-test, in which residuals were shuffled 10000 times globally. Gene probes with p < 0.05 after false discovery rate determination (FDR by Benjamini-Hochberg) were considered significantly changed. In cases of multiple probes per gene, the values from the most 3′ probe were used [27,28]. To determine differentially expressed genes a fold change cut-off of 2 fold was used. All microarray gene expression data have been deposited in NCBI’s Gene Expression Omnibus [85] and are accessible through GEO Series accession number GSE63054 [86].

### Quantitative reverse transcription-PCR

RNA was converted to cDNA using the iScript™ cDNA Synthesis Kit (BioRad, Veenendaal, The Netherlands) according to manufacturer’s instructions. Primers (Life Technologies; Additional file 2: Table S8) for specific *Bos taurus* mRNA templates [87] were designed using a Primer3 based platform [88]. Further in silico validation was performed by predicting PCR product folding structures using the Mfold web server [89-91]. For quantitative reverse transcription PCR (qRT-PCR) we used iQ™ SYBR® Green supermix on a MyiQ detection system (Biorad) in a 25 μl reaction volume with a final primer concentration of 400nM according to manufacturer’s instructions. To confirm specificity of primer pairs and establish melting temperatures (T_m_) a temperature gradient was performed ranging from 57.0°C – 65.3°C using a 4 times dilution series of cDNA from blastocyst samples. Reactions started with a 5 min enzyme activation cycle at 95°C continued with 45 cycles in which the first step was 20 sec denaturing at 95°C, followed by 30 sec at T_m_ (Additional file 2: Table S8) for annealing and the third step for 30 sec at 72°C for elongation. To generate a dissociation curve the reaction continued by increasing the temperature from 60°C to 98°C per 0.5°C for 15 sec each step. For expression analysis of the individual samples the primer specific optimal T_m_ was chosen (Additional file 2: Table S8) and the dissociation curve was generated with 1°C temperature increments per step until 98°C.

### Immunostaining

ICM, TE and blastocyst samples were collected and fixed in 4% paraformaldehyde (PFA) for 15 min and stored in 1% PFA at 4°C until further use. Samples were permeabilized in PBS + 10%FCS + 0.5% Triton X100 (Sigma Aldrich) for 30 min. Next, a-specific binding was blocked by incubating the samples in PBS + 10% FCS + 0.1% Triton X100 (PBST) for 1 hour before overnight incubation with primary antibodies rabbit anti-GATA6 (Santa Cruz;sc-9055;1:100), mouse anti-CDX2 (Biogenex; CDX2-88; 1:200) or mouse anti-NANOG (eBiosciences; 14-5768-82;1:250) at 4°C. Secondary antibody incubation for 1 hour with appropriate goat anti mouse Alexa647 or goat anti rabbit Alexa 488 dye (Invitrogen, Venlo, The Netherlands) and subsequent nuclear staining using DAPI (Sigma Aldrich) for 5 min preceded Vectashield (Brunschwig Chemie, Amsterdam, The Netherlands) mounting in Grace Bio-Labs SecureSeal™ imaging spacer (Sigma-Aldrich). All incubations were performed at room temperature unless stated otherwise.

Fluorescent images were obtained using an inverted semi-automated confocal microscope (SPE-II – DMI4000; Leica, Son, The Netherlands) and further analysed with Fiji software [92].

## Additional files

Additional file 1: Figure S1. Relative gene expression; additional data. Microarray expression data (A) were analysed for additional genes by qRT-PCR (B) in morula (purple), blastocyst (green), ICM (orange) and TE (blue). Normalization was performed with reference genes *RPL15*, *SDHA* and *YWHAZ*. Y-axis depicts relative mean expression to sample with highest expression set at 1 per gene. Bars with different letters are significantly different (p < 0.05) and error bars indicate standard deviation. Additional file 2: Table S1. Comparison of relative gene expression between blastocyst and morula. All genes differentially expressed (≥2-fold; p ≤ 0.05) between blastocyst and morula are ranked according to their relative expression. Positive numbers indicate enhanced expression in blastocysts and negative numbers indicate lower expression in blastocysts. **Table S2.** Relative expression of genes comparing ICM and TE. All genes differentially expressed (≥2-fold; p ≤ 0.05) between ICM and TE are ranked according to their relative expression. Positive numbers indicate higher expression in blastocysts and negative numbers indicate lower expression in blastocysts. **Table S3.** Singular enrichment analysis of ICM up-regulated genes. List of all enriched GO-terms. **Table S4.** Gene expression MAPK inhibited versus control ICMs. All genes differentially expressed (≥2-fold; p ≤ 0.05) between MAPK inhibited versus control ICMs are listed according to their relative expression. Positive numbers indicate higher expression in MAPK-inhibited ICMs and negative numbers have higher expression in control ICMs. **Table S5.** Singular enrichment analysis of up-regulated genes in the PD treated or in the control ICMs. GO-terms enriched for genes with up-regulated expression (PD), with down-regulated expression (DMSO) or in both analyses (PD &DMSO). **Table S6.** GO-terms enriched with ICM up-regulated, PD up- or PD down-regulated genes. Singular enrichment analysis with ICM up-regulated, PD up- or PD down-regulated genes (minimum of two genes in a GO-term and a false discovery rate ≤0.1). **Table S7.** Microarray sample composition. Numbers of embryos used for RNA extraction and subsequent microarray study are presented per sample. Note; Blastocyst sample 1 and 2 (B1 and B2) are identical to Blastocyst pool 1 and 2 (Bp1 and Bp2) respectively. **Table S8.** Primers used for qRT-PCR. Gene^r^ indicates a reference gene used for normalization. Additional file 3: Figure S2. Hierarchical tree graph of GO-terms enriched with ICM vs. TE up-regulated genes in the biological process GO-domain. All 406 up-regulated genes in the ICM versus TE comparison were analysed for their enrichment in biological processes by a singular enrichment analysis. Additional file 4: Figure S3. Relative gene expression in control and MAPK-inhibited inner cell mass; additional data. Microarray expression data (A) were confirmed by qRT-PCR (B) for 7 additional genes. MAPK-inhibited ICM (red) results are depicted relative to control ICM (green) expression. Normalization was performed with the reference genes *RPL15*, *SDHA* and *YWHAZ*. Error bars indicate standard deviation and significant differences are indicated by * p < 0.05; ** p < 0.005; *** p < 0.0005. Additional file 5: Figure S4. Effect of MAPK inhibition. Immunofluorescent staining for GATA6 (green) and NANOG (red) to determine protein distribution in control (upper panels) and MAPK-inhibited (lower panels) embryos. DAPI was used for nuclear staining.
